# Gestational Intermittent Hypoxia Enhances Mammary Stem Cells and Alters Tumor Phenotype in Adult Female Offspring

**DOI:** 10.3390/cells13030249

**Published:** 2024-01-29

**Authors:** Jaitri Joshi, Yue Xiong, Molly Kuhn, Abigail B. Radcliff, Tracy L. Baker, Jyoti J. Watters, Lisa M. Arendt

**Affiliations:** Department of Comparative Biosciences, School of Veterinary Medicine, University of Wisconsin-Madison, 2015 Linden Drive, Madison, WI 53706, USAtracy.baker@wisc.edu (T.L.B.); jjwatters@wisc.edu (J.J.W.)

**Keywords:** breast cancer, gestational intermittent hypoxia, mammary stem cells, sleep apnea, mammary gland

## Abstract

An adverse perinatal environment can increase long-term cancer risk, although the precise nature of associated perinatal triggers remain unknown. Sleep apnea is a common condition during pregnancy, characterized by recurrent cessations in breathing during sleep, and the potential consequences of sleep apnea during pregnancy as it relates to breast cancer risk in offspring have not been explored. To model sleep apnea, Sprague-Dawley dams were exposed during gestation to nightly intermittent hypoxia (GIH) or normoxia (GNx), and the mammary glands of female offspring were examined. GIH offspring demonstrated increased epithelial stem and progenitor cell populations, which are associated with diminished transforming growth factor beta (TGFβ) activity. Elevations in adipose tissue stem cells in the mammary gland were also identified in GIH offspring. In aging females, mammary tumors formed in GIH offspring. These tumors displayed a dramatic increase in stroma compared to tumors from GNx offspring, as well as distinct patterns of expression of stem cell-related pathways. Together, these results suggest that exposure to sleep apnea during pregnancy leads to lasting changes in the mammary glands of female offspring. Increased stem and progenitor cell populations as a result of GIH exposure could enhance long-term breast cancer risk, as well as alter the clinical behavior of resulting breast tumors.

## 1. Introduction

In 2021, breast cancer became the most common cancer type globally, and is the second most common cause of cancer death among American women [1]. Many factors that modulate breast cancer risk, such as age, family history, and reproductive factors such as age of first menarche or onset of menopause, are not modifiable. However, modifiable risk factors related to lifestyle are continuing to be identified, opening the field to new strategies for primary prevention. Approximately half of breast cancers develop in women who have no identifiable breast cancer risk factors other than gender (female) and age (over 40 years) [2], suggesting that there are also potentially unrecognized risk factors for breast cancer development.

Sleep apnea is a common disorder during pregnancy, characterized by recurrent cessations in breathing during sleep, which leads to pathologic drops in blood oxygen levels. These drops in blood oxygen levels are termed intermittent hypoxia (IH). Pregnancy can promote the initial development of sleep apnea or worsen pre-existing sleep apnea [3]. Sleep apnea prevalence in pregnancy is unclear due to a lack of screening [4,5], although estimates indicate that sleep apnea occurs in 15% of all pregnancies and >50% of high-risk pregnancies [3,4]. Sleep apnea often goes undetected during pregnancy because the questionnaires used for screening are unreliable [3,5]. Although sleep apnea is highly treatable, approximately 50% of pregnant women refuse sleep apnea testing, in part due to erroneous concerns that the continuous positive airway pressure device used for treatment will harm the fetus [5]. Importantly, breast cancer incidence is almost twice as high in women with sleep apnea [6,7], although this relationship is poorly appreciated and mechanistically not understood. While detrimental effects of sleep apnea during pregnancy on the mother and fetus have recently been identified [8], little is known about potential long-term consequences for the offspring. Using IH during gestation in rats to model sleep apnea, we have observed multiple systemic abnormalities in offspring rats including autism-relevant behavioral abnormalities, hypertensive responses, and altered respiratory control [9,10,11]. While many of these long-term physiologic changes occur in male offspring rats, questions remain regarding consequences for female offspring rats exposed to gestational IH (GIH).

To investigate whether exposure to sleep apnea could be an underlying long-term risk factor for breast cancer, we examined the mammary glands of female rat offspring whose mothers were exposed to GIH or gestational normoxia (GNx) during late gestation. We observed a long-term enrichment for mammary epithelial stem and progenitor cells in GIH offspring associated with diminished TGFβ activity. Further, GIH offspring had elevated numbers of multipotent stem cells within mammary adipose tissue. Aging GIH offspring developed mammary tumors with a significant increase in stromal cells, and gene expression analysis revealed distinct transcriptional programs related to stem and progenitor cells in tumors from GIH offspring as compared to those from GNx offspring. These results suggest that GIH causes long-term changes in the function of both epithelial and stromal cells in the mammary glands of female offspring rats. These functional changes could have significance for increased breast cancer risk and altered types of breast tumors that may form in daughters of women impacted by sleep apnea during pregnancy.

## 2. Materials and Methods

### 2.1. Intermittent Hypoxia Model

All studies were conducted with the approval of the Animal Care and Use Committee at the University of Wisconsin-Madison, per guidelines published by the NIH Guide for the Care and Use of Laboratory Animals (Animal Welfare Assurance No. D16-00239). Animals were housed in AAALAC-accredited facilities, and studies were conducted according to ARRIVE guidelines. Timed-pregnant Sprague-Dawley rats at gestational day (GD)9 (Charles River, Wilmington, MA, USA) were maintained on a 12:12 h light/dark cycle with water and food provided ad libitum, then randomized to either GIH or GNx conditions. From GD10 until GD21, dams were exposed to 2-min cycles of 10.5% O_2_ hypoxic or intermittent room air (21% O_2_; normoxia) alternating 15 times per hour for 8 h [9]. The dams were returned to normal cages prior to parturition to prevent the direct exposure of offspring to hypoxic conditions. Age at puberty was determined by the day of vaginal opening. Vaginal cytology was performed for 2 weeks following the date of vaginal opening to determine estrous cycle length. Following humane euthanasia, the second, third, and fourth mammary glands were collected from 8 and 20-week-old female offspring. A cohort of offspring were housed either until tumors spontaneously formed in any of the mammary glands, or until the offspring reached 24 months of age.

### 2.2. Mammary Whole Mounts

The left fourth mammary glands were fixed in 10% neutral buffered formalin for 48 h, then stained with carmine alum. The tissue was treated with graded ethanol, defatted using xylenes, then stored in glycerol. Mammary whole-mounts were imaged with IS-Capture software (version 3.9.0.601, Tucsen Photonics) using a dissecting microscope (AMScope, Irvine, CA, USA, IN-300T-FL). Ductal branching was counted using ImageJ software version 1.52a (ImageJ, NIH, Bethesda, MD, USA). The length and width of ductal growth from the nipple was measured, and the percentage area of ductal outgrowth was calculated using the area of the mammary fat pad.

### 2.3. Histology

All slides were blinded prior to staining and quantification. Five representative areas of adipose tissue on hematoxylin and eosin-stained slides were imaged using a Nikon Eclipse E600 microscope (Nikon, Melville, NY, USA). The diameters of 10 adipocytes/image were measured with the ruler tool (NIS-Elements software, Basic Research (BR) version). For immunohistochemistry, tissue sections were deparaffinized, rehydrated in graded alcohol, and then underwent antigen retrieval in 0.01 M citrate buffer (pH = 6.0). Tissue sections were incubated with anti-rat Ki67 antibodies (1:250 dilution; Abcam, Waltham, MA, USA, ab157301), phosphorylated SMAD2 (1:250 dilution; Fisher Scientific, Waltham, MA, USA, 44-244G), or CD68 monoclonal antibodies (KP1; 1:250 dilution; Invitrogen, Carlsbad, CA, USA, MA5-13324) diluted in 5% fish gelatin/TBST. Tissue sections were incubated with a 1:250 dilution of biotinylated anti-rabbit (1:250, BA-1000) or anti-mouse (1:250, MKB-2225) secondary antibodies from Vector Laboratories (Newark, CA, USA). The samples were counterstained with hematoxylin. Five ducts/slide were imaged, 100 cells/image were quantified, and the percentage of positive nuclei were quantified.

### 2.4. Collagen Quantification

Paraffin-embedded tissue sections were stained for picrosirius red as described [12]. Stained tissue was imaged using a Nikon Eclipse E600 microscope and NIS-Elements software. Five ducts were imaged for each slide. Collagen was quantified by subtracting autofluorescence from the picrosirius red staining identified with the 532 nm laser, and the area of collagen staining was quantified as described [13].

### 2.5. Isolation of Mammary Epithelial and Stromal Cells

Rat mammary tissue was minced, then digested overnight at 37 °C as described [14]. Clusters of epithelial cells were settled for 20 min, and then underwent centrifugation for 5 min at 8× *g*. The supernatant containing the adipose stromal cells was removed and pelleted. The mammary epithelial and stromal cells were treated with red blood cell lysis buffer (ACK Lysing Buffer, Lonza, Walkersville, MD, USA, 10-548E) and washed with 5% calf serum in PBS. The adipose stromal cells were plated in DMEM with 10% fetal bovine serum, gentamicin sulfate (0.5 mg/mL, Fisher Scientific, AC455310010) [15], and 1% antibiotic/antimycotic (Corning, Corning, NY, USA, 30-004-CI), and adherent cells were grown for no more than 3 passages. The mammary epithelial cells were dissociated to single cells for progenitor assays as described [16]. Collagenase digested tumors were plated for 1 h in DMEM with 10% fetal bovine serum and 1% antibiotic/antimycotic to deplete stromal cells prior to RNA extraction from epithelial cells.

### 2.6. Progenitor Assays

To assess mammosphere forming ability, 5000 mammary epithelial cells from four rats/group were plated in triplicate on 24-well ultra-low-attachment plates (Corning, 3473) in 500 µL as described [14]. Mammospheres formed for 7 days at 37 °C with 5% CO_2_ and were counted and imaged using an inverted microscope. To assess secondary mammosphere formation, primary mammospheres were collected, trypsinized, and re-plated onto ultra-low-attachment plates for 7 days.

To assess colony forming abilities on tissue culture plates, viable cells from single-cell suspensions from four rats/group were plated at 7500 cells/plate in duplicate on 35 mm^2^ tissue culture plates coated with 50 µg/mL rat tail collagen (Corning, 354236) as described [14]. Following colony formation, plates were fixed with ice-cold methanol for 10 min, then stained with crystal violet. Colonies were counted using a light microscope.

### 2.7. Differentiation Assays

To assess differentiation potential, 1 × 10^5^ adipose stromal cells were plated on six-well plates with DMEM and supplemented with 10% FBS and 1% antibiotic/antimycotic until confluent. For bone differentiation, media was supplemented with 0.01 M β-glycerol phosphate (MilliporeSigma, St. Louis, MO, USA, 50020) and 100 mM ascorbic acid (Sigma, A4544) and was replaced every 2–3 days. On day 21, cells were collected for RNA extraction or stained with alizarin red. Alizarin red stain was extracted and quantified by measuring absorbance at 405 nm (Bio Tek, Shoreline, WA, USA, 7091000). For adipocyte differentiation, media was supplemented with 0.5 µM dexamethasone (Sigma, D4902), 0.5 mM 3-isobutyl methyl xanthine (Sigma, I7018), 0.5 µg/mL insulin (Sigma, I5500), and 50 µM indomethacin (Fisher Scientific, AC458030050) for 7 days. After 7 days, media was supplemented with 0.5 µg/mL insulin (Sigma, I0516) and was replaced every 2–3 days. On day 21, cells were collected for RNA extraction or stained with oil red O. Oil red O stain was extracted and quantified by measuring absorbance at 510 nm.

### 2.8. Gene Expression Analyses

RNA was isolated using TRIzol (ThermoFisher, Waltham, MA, USA, 15596018) following the manufacturer’s protocol. RNA cleanup was performed using an RNeasy Plus Micro Kit (Qiagen, Germantown, MD, USA, 74034) and quantified using a NanoDrop 1000 Spectrophotometer (ThermoFisher, ND-1000). cDNA was synthesized using the High-Capacity cDNA Reverse Transcription Kit (Applied Biosciences, Livermore, CA, USA, 4368814), and qRT-PCR was performed using iTaq Universal SYBR Green Supermix (Bio-Rad, Hercules, CA, USA, 172521) and the CFX Connect Optics Module (Bio-Rad). Data was normalized to 18S ribosomal RNA and analyzed using the ΔΔCq method. Primers are found in Table S1.

RNA isolated from primary epithelial cells or tumor cells was amplified using the RT^2^ PreAMP cDNA Synthesis Kit (Qiagen, 330451) with RT^2^ PreAMP cDNA synthesis primer Mix (Qiagen, 047Z) or RT^2^ First Strand Kit (330401). Gene expression was quantified using RT^2^ Profiler PCR Rat Stem Cell Signaling Arrays (Qiagen, PARN-047Z) with RT^2^ SYBR Green qPCR Mastermix (Qiagen, 330502), and analysis was completed using GeneGlobe Analysis Software (Qiagen).

### 2.9. Statistical Analyses

The results between the two groups were compared using an unpaired *t*-test, and an F test was used to compare the variances. For differentiation assays, data were analyzed using one-way ANOVA with Tukey’s multiple comparison test. Data are represented using mean ± s.e.m. All statistics were calculated using GraphPad Prism v 9.3.1. Statistical significance was determined at a significance level of *p* = 0.05.

## 3. Results

### 3.1. Delayed Development of Mammary Glands in GIH Female Offspring

**Figure 1:**
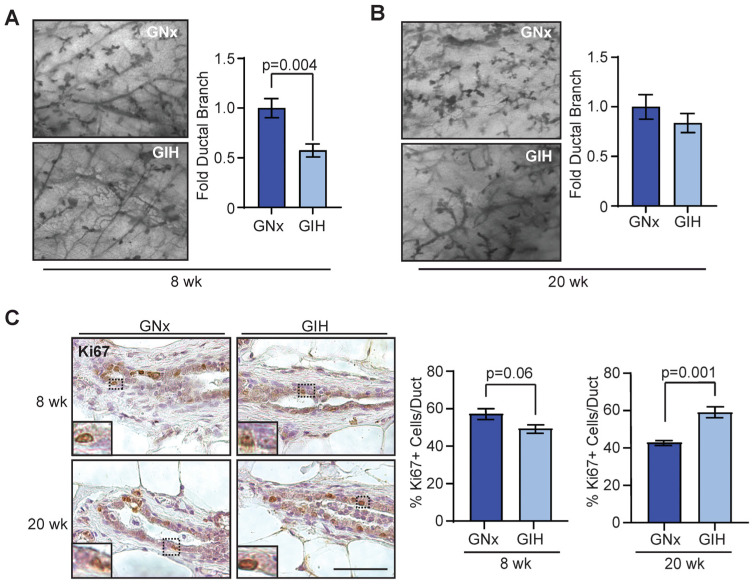
Delayed mammary gland development in GIH offspring female rats. Quantification of ductal branching within the mammary fat pads in normoxic (GNx) and hypoxic (GIH) offspring at 8 weeks ((**A**), *n* = 6 rats/group) and 20 weeks ((**B**), *n* = 6 GNx, *n* = 5 GIH rats). (**C**) Ki67 expression in ducts from mammary tissue from 8 weeks (*n* = 6) and 20 weeks (*n* = 6 GNx, *n* = 5 GIH) offspring rats. Dotted lines indicate area of magnification for inset image. Magnification of images (**A**,**B**) 40×. Magnification bars: (**A**), 0.5 mm; (**C**), 100 µm.

Dams were exposed to either GIH or GNx during their typical period of heightened sleep, similar to humans with sleep apnea [17]. Since the timing of GIH induction was during the period of fetal mammary gland development [18], we hypothesized that exposure to GIH during pregnancy may lead to alterations in mammary gland development of female offspring rats. We examined mammary whole mounts from rats after puberty (8 weeks) and mature rats (20 weeks). We did not observe any differences in the percentage of mammary glands filled with ducts (Figure S1A,B) or alveolar budding (Figure S1C,D). However, at 8 weeks, GIH offspring had significantly reduced ductal branching compared to GNx offspring (*p* = 0.004, Figure 1A), which was resolved by the 20-week age time point (Figure 1B).

Mammary gland development is orchestrated by ovarian hormones. No significant differences were observed in offspring at the start of puberty, although the GIH offspring showed more variability than GNx offspring (*p* = 0.003, F-test, Figure S1E). Further, no significant differences were observed in the length of time that the offspring spent in estrus or diestrus during the estrus cycle over a 2-week period (Figure S1F,G). Previous data has shown that serum levels of 17β-estradiol were not significantly different between offspring at 12 weeks of age [9]. These data suggest that GIH offspring do not have significant differences in ovarian function that impact mammary gland development.

We examined changes in mammary epithelial cell proliferation over time. While proliferation marker Ki67 expression was modestly reduced in 8-week-old GIH offspring compared to GNx offspring (*p* = 0.06), at 20 weeks, GIH offspring demonstrated significantly increased Ki67 expression compared to GNx females (*p* = 0.001, Figure 1C). This suggests that GIH induces long-term increases in proliferation in the mammary epithelial cells of offspring.

### 3.2. Enhanced Mammary Stem/Progenitor Activity in GIH Offspring

**Figure 2:**
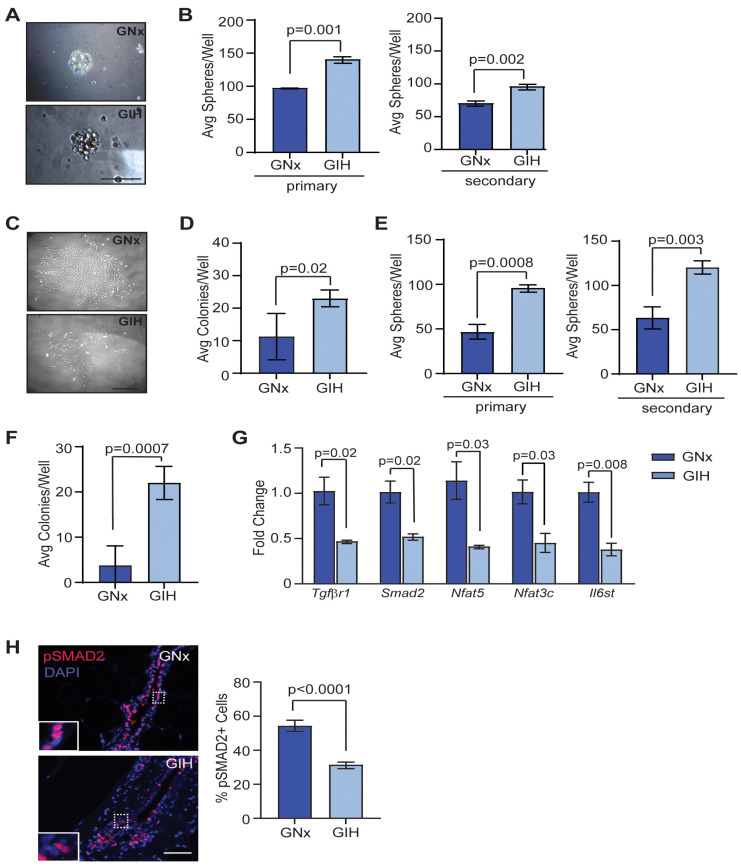
Increased mammary stem and progenitor cell activity in GIH offspring rats. (**A**) Representative image of mammospheres. (**B**) Average primary and secondary mammospheres per well for 8-week-old GNx and GIH offspring rat mammary glands (*n* = 4 rats/group). (**C**) Representative image of colonies. (**D**) Average mammary epithelial cell colonies per well (*n* = 4 rats/group). (**E**) Average primary and secondary mammospheres per well for 20-week-old GNx and GIH offspring rat mammary glands (*n* = 4 rats/group). (**F**) Average mammary epithelial cell colonies per well (*n* = 4 rats/group). (**G**) Differences in gene expression in mammary epithelial cells isolated from mammary glands of GNx and GIH offspring quantified using qRT-PCR arrays (*n* = three rats/group). (**H**) Representative images and quantification of phosphorylated SMAD2 (pSMAD2) staining in rat mammary ducts (*n* = 5/group). Dotted lines indicate area of magnification for inset image. Magnification bar = 100 µm.

The most common types of breast cancer are thought to arise from mutations or epigenetic modifications in epithelial stem and progenitor cells, which may be acquired at key phases of rapid cell division, such as during in utero development or during puberty [19,20]. To investigate how GIH exposure impacts mammary stem and progenitor cells, we isolated mammary epithelial cells from GIH and GNx offspring and plated the cells in limiting dilution to measure mammospheres and colony formation. At 8 weeks, mammary epithelial cells from GIH offspring had formed significantly greater numbers of both primary and secondary mammospheres (Figure 2A,B). No differences were observed in the shape or size of the mammospheres. When plated on adherent plates coated in collagen, mammary epithelial cells isolated from GIH offspring demonstrated significantly enhanced colony formation (*p* = 0.02, Figure 2C,D). Although the colonies generated from the primary epithelial cells from each rat were variably sized, no differences were observed in colony size based on group. At 20 weeks, epithelial cells from GIH offspring also developed significantly more primary and secondary mammospheres (Figure 2E) as well as adherent colonies (*p* = 0.0007; Figure 2F).

To identify potential mechanisms for increased stem and progenitor cell activity, we isolated mammary epithelial cells from 20-week-old GNx and GIH offspring and examined gene expression using stem cell pathway-focused qRT-PCR arrays. Surprisingly, we did not observe any genes that were significantly upregulated in mammary epithelial cells from GIH offspring compared to those from GNx offspring. Genes that were downregulated in mammary epithelial cells from GIH offspring included *Tgfbr1*, a downstream mediator of TGFβ signaling, *Smad2*, as well as *Nfat5*, *Nfat3c*, and *Il6st* (Figure 2G). Since TGFβ is a major negative regulator of epithelial cell stem and progenitor activity in the mammary gland [21,22], we examined phosphorylation of SMAD2 to assess TGFβ signaling activity [23]. We observed a significant decrease in nuclear localization of phosphorylated SMAD2 within epithelial cells of GIH offspring compared to GNx offspring (*p* < 0.0001, Figure 2H). Together, these data suggest that GIH exposure increases long-term mammary stem and progenitor activity through downregulation of the TGFβ signaling pathway.

### 3.3. Microenvironmental Changes Observed in Mammary Glands of GIH Offspring

**Figure 3:**
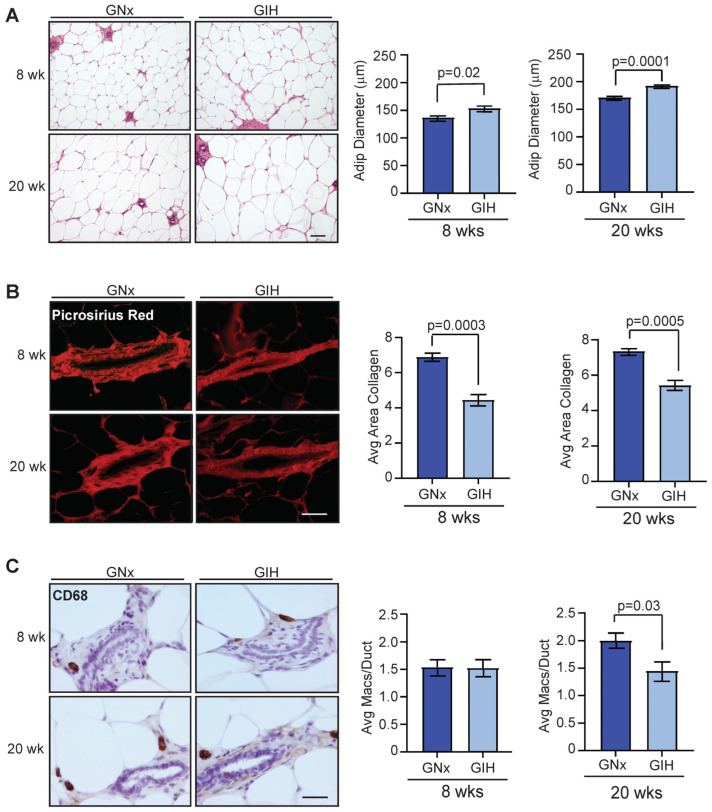
Alterations in stromal cell populations in GIH offspring rats. (**A**) Adipocyte diameters in mammary tissue from offspring rats at 8 weeks (*n* = 6 rats/group) and 20 weeks (*n* = 6 GNx, *n* = 5 GIH). (**B**) Average area of collagen deposition surrounding ducts in offspring rat tissue at 8 weeks (*n* = 6/group) and 20 weeks (*n* = 6 GNx, *n* = 5 GIH). (**C**) CD68^+^ macrophages surrounding ducts in mammary tissue were quantified from GNx and GIH offspring rats at 8 weeks (*n* = 6 rats/group) and 20 weeks (*n* = 6 GNx, *n* = 5 GIH). Magnification bar = 100 µm.

Recent work in a mouse model of GIH demonstrated that the exposure of pregnant mice to IH during pregnancy altered the function of adipose tissue of male offspring, leading to metabolic dysfunction [24]. To examine how exposure to GIH impacts cell types in the stroma of mammary glands, we measured the diameter of adipocytes found within the mammary tissue of GNx and GIH offspring. At both 8 and 20 weeks of age, adipocyte diameters were significantly larger in the mammary glands of GIH offspring compared to GNx offspring (Figure 3A). Interestingly, we observed that GIH offspring had reduced collagen deposition surrounding mammary ducts at both 8 and 20-week time points (Figure 3B). We also examined CD68^+^ macrophages surrounding ducts, which have been shown to promote extracellular matrix remodeling [25]. At 8 weeks, no significant difference was observed in the number of CD68^+^ macrophages surrounding mammary ducts (Figure 3C). However, at 20 weeks, we observed significantly reduced numbers of CD68^+^ macrophages surrounding mammary ducts in GIH offspring (*p* = 0.03, Figure 3C). Together, these results suggest that GIH offspring also have lasting changes in the mammary stroma surrounding the epithelial cells.

### 3.4. Increased Differentiation Potential in Adipose-Derived Stromal Cells from GIH Offspring

**Figure 4:**
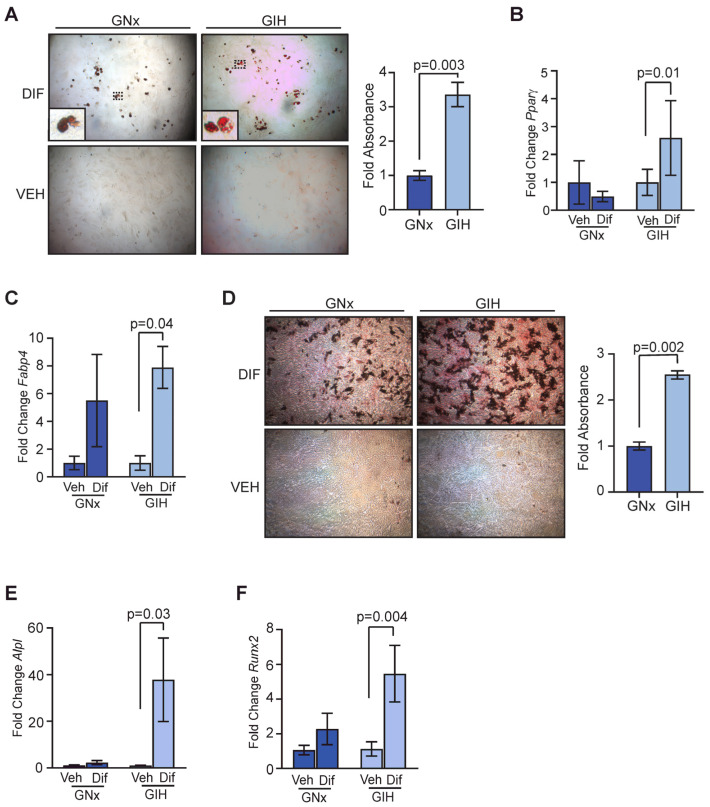
Increased differentiation potential of adipose-derived stromal cells from GIH offspring rats. (**A**) Representative images of oil red O staining and quantification of adipose-derived stromal cells from 8 week old rats exposed to adipogenic media (Dif) or vehicle controls (Veh; *n* = 4 rats/group). Dotted lines indicate area of magnification for inset image. Expression of *Pparγ* (**B**) and *Fabp4* (**C**) from adipose-derived stromal cells exposed to adipogenic media or vehicle controls quantified using qRT-PCR (*n* = 4 rats/group). (**D**) Representative images of alazarin red staining and quantification of adipose-derived stromal cells from 8 week old rats exposed to bone differentiation media (Dif) or vehicle controls (Veh; *n* = 4 rats/group). Expression of *Alpl* (**E**) and *Runx2* (**F**) from adipose-derived stromal cells exposed to bone differentiation media or vehicle controls quantified using qRT-PCR (*n* = 4 rats/group). Magnification bar = 100 µm.

Adipose tissue, including subcutaneous adipose tissue in the mammary gland, contains a population of stem cells that have the ability to differentiate into mesenchymal cell lineages in response to different stimuli [26]. Given the changes in the mammary stroma that we observed in GIH offspring, we investigated whether there were also differences in this stem cell population between GNx and GIH offspring. We expanded the adipose-derived stromal cells from 20-week-old offspring in vitro. In response to adipogenic media, adipose-derived stromal cells from GIH offspring had significantly increased lipid droplets (*p* = 0.003, Figure 4A). Further, the expression of markers of adipocyte differenti ation, *Pparγ* (*p* = 0.01) and *Fabp4* (*p* = 0.04), were significantly elevated in differentiated stromal cells from GIH offspring compared to undifferentiated control cells (Figure 4B,C). Similarly, in response to bone differentiation media, adipose-derived stromal cells from GIH offspring had significantly increased deposition of bone matrix (*p* = 0.002, Figure 4D). The expression of markers of bone differentiation *Alpl* (*p* = 0.03, Figure 4E) and *Runx2* (*p* = 0.004, Figure 4F) was significantly higher in adipose-derived stromal cells from GIH offspring compared to undifferentiated controls.

To examine contaminating cell types, we quantified the gene expression of epithelial cell marker CD24, endothelial cell marker CD31, and immune cell marker CD45. No significant differences were observed between stromal cells from GNx and GIH offspring (Figure S2A–C). We also observed similar expression levels of stromal cell markers in the adipose-derived stromal cells from GNx and GIH offspring (Figure S2D). These results suggest that similar to the epithelial cell compartment in the mammary gland, adipose stem cells are enhanced in the mammary glands of GIH offspring.

### 3.5. Fibrous Tumors Enhanced in Mammary Glands of GIH Offspring

Sprague-Dawley rats develop spontaneous mammary tumors during aging [27]. Although no significant differences were observed in latency to tumor formation between GNx and GIH offspring (Figure S3A), GIH offspring developed mammary tumors with a mildly higher frequency, with an incidence of mammary tumors in 12 out of 27 rats (44%), while GNx offspring developed tumors with an incidence of 10 out of 34 rats (29%) (Figure S3B). Aging Sprague-Dawley rats are susceptible to pituitary tumors, which may enhance mammary tumor formation through elevated circulating prolactin levels [28]. While many offspring that developed mammary tumors in both treatment groups also developed pituitary tumors (Figure S3C), treatment did not significantly affect the incidence of pituitary tumors, and the presence of pituitary tumors did not alter mammary tumor latency (Figure S3D).

**Figure 5:**
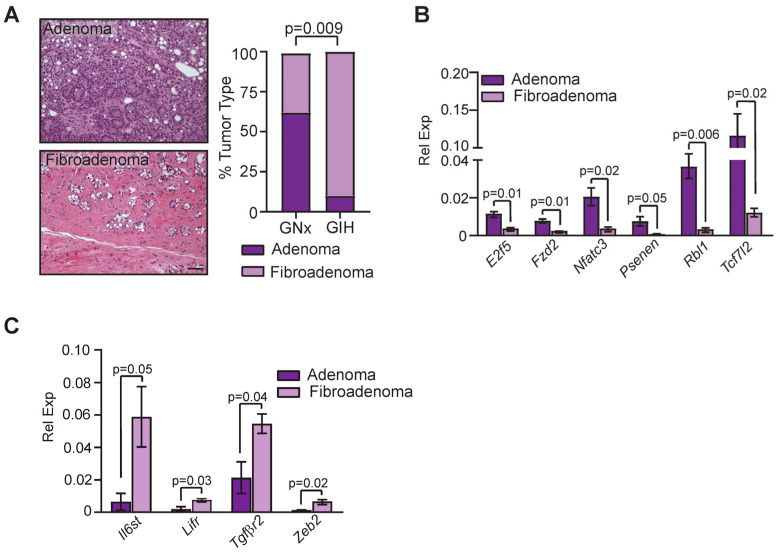
Mammary fibroadenomas enriched in GIH offspring rats. (**A**) Tumor histotypes in aging GNx and GIH offspring rats (*n* = 10 GNx, *n* = 12 GIH). (**B**) Genes with upregulated expression in adenomas from offspring rats quantified using qRT-PCR arrays (*n* = 3 rats/group). (**C**) Genes with upregulated expression in fibroadenomas from offspring rats quantified using qRT-PCR arrays (*n* = 3 rats/group). Magnification bar = 100 µm.

Tumors that formed in the mammary glands of aging offspring of both treatment groups were well-differentiated adenomas and fibroadenomas that expressed estrogen receptor alpha (ERα) at similar levels (Figure S3E). We observed a significant shift to favoring the formation of fibroadenomas, which contain a large component of stromal cells, in GIH offspring compared to GNx offspring (*p* = 0.009, Figure 5A). To identify how stem cell signaling pathways were altered between adenomas and fibroadenomas, we isolated epithelial cells from tumors and examined gene expression using qRT-PCR arrays. Adenomas had significantly increased expression of transcription factors *E2f5* and *Tcf712*, Wnt family receptor *Fzd2*, transcriptional regulator *Nfatc3*, regulator of the gamma secretase complex *Psenen*, and *Rb* (Figure 5B). In contrast, fibroadenomas had elevated expression of *Il6st*, *Lifr*, *Tgfβr2*, and transcription factor *Zeb2* (Figure 5C). Together these data suggest that GIH exposure alters the phenotype of mammary tumors, and the tumors in mammary glands of GIH offspring are enriched for different stem cell pathways than tumors from GNx offspring.

## 4. Discussion

Sleep apnea during pregnancy is a risk factor for multiple complications affecting both the mother and fetus [29]. Little is known about the impact of sleep apnea during pregnancy on the long-term health of the offspring. Using a rat model of sleep apnea during pregnancy, we observed transient developmental delays in mammary gland ductal branching. Although the differences in ductal branching resolved, we observed lasting increases in stem and progenitor activity in both mammary epithelial cells and adipose-derived stem cells in the mammary gland in female offspring. Changes in these stem cell compartments may promote differences in the types of mammary tumors that develop, as GIH offspring formed fibroadenomas with a significantly enriched stromal compartment and distinct gene expression of stem cell-related pathways. Together, these results suggest that GIH exposure has long-lasting effects on the mammary gland that could impact breast cancer development later in life.

Multiple signaling pathways regulate mammary epithelial stem cell activity. In mammary epithelial cells from GIH offspring, we observed significantly reduced expression of *Tgfrβ1* and downstream regulator *Smad2* compared to GNx offspring. TGFβ1 is a negative regulator of mammary epithelial stem/progenitor activity [21,22], and reduced activity in this pathway may result in enhanced mammary stem and progenitor activity. Less is known about the function of Nfat5 in the mammary gland. In the intestines, Nfat5 represses canonical Wnt signaling to regulate stem cell differentiation [30], and reduced Nfat5 expression may enhance stem cell activity in mammary epithelial cells of GIH offspring through a Wnt-mediated mechanism. We also observed diminished expression of *Nfatc3* and *Il6st*, which function as tumor suppressor genes in the mammary gland [31,32]. Interestingly, we did not observe any significantly upregulated genes associated with stem and progenitor cell activity. This may be indicative of more global changes in gene expression through methylation, as has been observed in adipose tissue of male GIH offspring [24,33].

Adipose-derived stromal cells from GIH offspring also demonstrated increased ability to differentiate into mesenchymal lineages in vitro. This ability for multipotent differentiation is consistent with increased adipose stem cells within the mammary gland. Given the shift toward fibroadenomas in the mammary glands of aging GIH offspring, it is possible that elevated adipose stem cells played a role in the growth of these tumors. During tumorigenesis, adipose stem cells have been shown to differentiate into cancer-associated fibroblasts in multiple types of cancer [34]. We also observed a significant increase in *Tgfβr2* expression within fibroadenomas that was not observed in adenomas. This data is suggestive of elevated TGFβ signaling within the tumor microenvironment, and TGFβ is a major promoter of tumor fibrosis [35]. Increased cancer-associated fibroblasts and collagen deposition has been associated with a poor prognosis for breast tumors in human studies [36,37].

Women with sleep apnea have a significantly higher risk for breast cancer [6,7]. The mechanisms for this increased risk are still under investigation. While women with sleep apnea have periods of reduced blood oxygen saturation, which could lead to enhanced expression of hypoxia-associated genes, evidence suggests that maternal hypoxia due to gestational sleep apnea may not lead to fetal hypoxia [38]. Sleep apnea also induces a chronic inflammatory state, causing elevated levels of circulating cytokines [39]. Sleep apnea has also been shown to induce global epigenetic changes in humans [40] as well as animal models [41], although the mechanism promoting epigenetic remodeling is unknown. Adverse events that occur during fetal development can induce persistent epigenetic modifications that impact long-term health [42,43]. Long-term changes in epigenetic programming could impact not only breast cancer risk [44], but also treatment responses in breast tumors that develop [45,46].

Emerging evidence in human clinical studies demonstrates that many of the observations that we have made in rodent offspring following GIH exposure also occur in children whose mothers had sleep apnea during pregnancy [47,48,49]. However, before links between maternal sleep apnea and increased disease risk in offspring can be systematically investigated in humans, animal models must provide the justification. Ethical concerns preclude allowing human pregnancies with sleep apnea to proceed without medical intervention. Interventions include using a continuous positive airway pressure device to eliminate the periods of sleep apnea during sleep so that both the mother and the developing fetus are protected from the pathological effects of hypoxia. In humans, the multifactorial aspects of sleep apnea, such as body weight, genetics, and sleep rhythms, are not experimentally separable [50]. Rodent GIH studies are a necessary strength to detangle the complex mechanisms and interactions whereby sleep apnea alters fetal physiology into adulthood.

## 5. Conclusions

Sleep apnea is a common disorder in pregnant women. Both screening and testing opportunities for sleep apnea during pregnancy are limited, although treatment with continuous positive airway pressure during sleep ameliorates sleep apnea incidence and pathological sequelae. Although pregnancy complications due to sleep apnea have been identified, little is known about the long-term impact of sleep apnea on offspring. Here we show that GIH exposure leads to lasting increases in mammary stem and progenitor cells in both the epithelium and stromal compartments. Elevated numbers of epithelial stem and progenitor cells could enhance breast cancer risk over time, particularly with exposure to environmental carcinogens or unhealthy lifestyle choices. Improving interventions to diagnose and treat sleep apnea during pregnancy could impact long-term breast cancer risk for offspring.

## Data Availability

All data generated or analyzed during this study are included in this published article.

## Supplementary Materials

The following supporting information can be downloaded at: https://www.mdpi.com/article/10.3390/cells13030249/s1, Figure S1: Mammary gland development, timing of puberty, and estrus cycle in offspring rats; Figure S2: Characterization of adipose-derived stromal cells from mammary glands of GNx and GIH offspring rats; Figure S3: Latency of tumors from GNx and GIH aging offspring rats; Table S1: Primers used for quantitative RT-PCR analyses.

## References

[1] American Cancer Society (2023). Cancer Facts and Figures 2023.

[2] World Health Organization (2021). Obesity and Overweight. https://www.who.int-news-room/fact-sheets/detail/obesity-and-overweight.

[3] Johns E.C., Denison F.C., Reynolds R.M. (2020). Sleep disordered breathing in pregnancy: A review of the pathophysiology of adverse pregnancy outcomes. Acta Physiol..

[4] Young T., Peppard P.E., Gottlieb D.J. (2002). Epidemiology of obstructive sleep apnea: A population health perspective. Am. J. Respir. Crit. Care Med..

[5] Antony K.M., Jacobson N.M., Rice L., Wiedmer A.M., Mourey H., Bazalakova M.H. (2021). Obstructive sleep apnea in pregnancy: Early lessons from our sleep pregnancy clinic. WMJ.

[6] Chang W.P., Liu M.E., Chang W.C., Yang A.C., Ku Y.C., Pai J.T., Lin Y.W., Tsai S.J. (2014). Sleep apnea and the subsequent risk of breast cancer in women: A nationwide population-based cohort study. Sleep Med..

[7] Yap D.W.T., Tan N.K.W., Tan B.K.J., Teo Y.H., Tan V.K.M., See A., Toh S.T. (2022). The association of obstructive sleep apnea with breast cancer incidence and mortality: A systematic review and meta-analysis. J. Breast Cancer.

[8] Warland J., Dorrian J., Morrison J.L., O’Brien L.M. (2018). Maternal sleep during pregnancy and poor fetal outcomes: A scoping review of the literature with meta-analysis. Sleep Med. Rev..

[9] Song R., Mishra J.S., Dangudubiyyam S.V., Antony K.M., Baker T.L., Watters J.J., Kumar S. (2022). Gestational intermittent hypoxia induces sex-specific impairment in endothelial mechanisms and sex steroid hormone levels in male rat offspring. Reprod. Sci..

[10] Johnson S.M., Randhawa K.S., Epstein J.J., Gustafson E., Hocker A.D., Huxtable A.G., Baker T.L., Watters J.J. (2018). Gestational intermittent hypoxia increases susceptibility to neuroinflammation and alters respiratory motor control in neonatal rats. Respir. Physiol. Neurobiol..

[11] Vanderplow A.M., Kermath B.A., Bernhardt C.R., Gums K.T., Seablom E.N., Radcliff A.B., Ewald A.C., Jones M.V., Baker T.L., Watters J.J. (2022). A feature of maternal sleep apnea during gestation causes autism-relevant neuronal and behavioral phenotypes in offspring. PLoS Biol..

[12] Chamberlin T., Clack M., Silvers C., Kuziel G., Thompson V., Johnson H., Arendt L.M. (2020). Targeting obesity-induced macrophages during preneoplastic growth promotes mammary epithelial stem/progenitor activity, DNA damage, and tumor formation. Cancer Res..

[13] Kuziel G., Moore B.N., Haugstad G.P., Arendt L.M. (2023). Fibrocytes enhance mammary gland fibrosis in obesity. FASEB J..

[14] Tovar E.A., Sheridan R., Essenburg C.J., Dischinger P.S., Arumugam M., Callaghan M.E., Graveel C.R., Steensma M.R. (2020). Dissecting the rat mammary gland: Isolation, characterization, and culture of purified mammary epithelial cells and fibroblasts. Bio Protoc..

[15] El Mouedden M., Laurent G., Mingeot-Leclercq M.P., Tulkens P.M. (2000). Gentamicin-induced apoptosis in renal cell lines and embryonic rat fibroblasts. Toxicol. Sci..

[16] Chamberlin T., D’Amato J.V., Arendt L.M. (2017). Obesity reversibly depletes the basal cell population and enhances mammary epithelial cell estrogen receptor alpha expression and progenitor activity. Breast Cancer Res..

[17] Lim D.C., Brady D.C., Po P., Chuang L.P., Marcondes L., Kim E.Y., Keenan B.T., Guo X., Maislin G., Galante R.J. (2015). Simulating obstructive sleep apnea patients’ oxygenation characteristics into a mouse model of cyclical intermittent hypoxia. J. Appl. Physiol..

[18] Filgo A.J., Foley J.F., Puvanesarajah S., Borde A.R., Midkiff B.R., Reed C.E., Chappell V.A., Alexander L.B., Borde P.R., Troester M.A. (2016). Mammary gland evaluation in juvenile toxicity studies: Temporal developmental patterns in the male and female Harlan Sprague-Dawley rat. Toxicol. Pathol..

[19] Ginestier C., Wicha M.S. (2007). Mammary stem cell number as a determinate of breast cancer risk. Breast Cancer Res..

[20] Terry M.B., Michels K.B., Brody J.G., Byrne C., Chen S., Jerry D.J., Malecki K.M.C., Martin M.B., Miller R.L., Neuhausen S.L. (2019). Environmental exposures during windows of susceptibility for breast cancer: A framework for prevention research. Breast Cancer Res..

[21] Kordon E.C., McKnight R.A., Jhappan C., Hennighausen L., Merlino G., Smith G.H. (1995). Ectopic TGF beta 1 expression in the secretory mammary epithelium induces early senescence of the epithelial stem cell population. Dev. Biol..

[22] Boulanger C.A., Smith G.H. (2001). Reducing mammary cancer risk through premature stem cell senescence. Oncogene.

[23] Feng X.-H., Derynck R. (2005). Specificity and versatility in TGF-β signaling through SMADS. Ann. Rev. Cell Dev. Biol..

[24] Khalyfa A., Cortese R., Qiao Z., Ye H., Bao R., Andrade J., Gozal D. (2017). Late gestational intermittent hypoxia induces metabolic and epigenetic changes in male adult offspring mice. J. Physiol..

[25] Wang Y., Chaffee T.S., LaRue R.S., Huggins D.N., Witschen P.M., Ibrahim A.M., Nelson A.C., Machado H.L., Schwertfeger K.L. (2020). Tissue-resident macrophages promote extracellular matrix homeostasis in the mammary gland stroma of nulliparous mice. eLife.

[26] Si Z., Wang X., Sun C., Kang Y., Xu J., Wang X., Hui Y. (2019). Adipose-derived stem cells: Sources, potency, and implications for regenerative therapies. Biomed. Pharmacother..

[27] Davis R.K., Stevenson G.T., Busch K.A. (1956). Tumor incidence in normal Sprague-Dawley female rats. Cancer Res..

[28] Meites J. (1982). Changes in neuroendocrine control of anterior pituitary function during aging. Neuroendocrinology.

[29] Facco F.L., Chan M., Patel S.R. (2022). Common sleep disorders in pregnancy. Obstet. Gynecol..

[30] Wang Q., Zhou Y., Rychahou P., Liu C., Weiss H.L., Evers B.M. (2013). NFAT5 represses canonical Wnt signaling via inhibition of β-catenin acetylation and participates in regulating intestinal cell differentiation. Cell Death Dis..

[31] Lee H., Chouinard L., Bonin M., Michel R.N. (2005). NFATc3 deficiency may contribute to the development of mammary gland adenocarcinoma in aging female mice. Mol. Carcinog..

[32] Jia R., Weng Y., Li Z., Liang W., Ji Y., Liang Y., Ning P. (2021). Bioinformatics analysis identifies IL6ST as a potential tumor suppressor gene for triple-negative breast cancer. Reprod. Sci..

[33] Badran M., Yassin B.A., Lin D.T.S., Kobor M.S., Ayas N., Laher I. (2019). Gestational intermittent hypoxia induces endothelial dysfunction, reduces perivascular adiponectin and causes epigenetic changes in adult male offspring. J. Physiol..

[34] Jotzu C., Alt E., Welte G., Li J., Hennessy B.T., Devarajan E., Krishnappa S., Pinilla S., Droll L., Song Y.H. (2011). Adipose tissue derived stem cells differentiate into carcinoma-associated fibroblast-like cells under the influence of tumor derived factors. Cell. Oncol..

[35] Chung J.Y., Chan M.K., Li J.S., Chan A.S., Tang P.C., Leung K.T., To K.F., Lan H.Y., Tang P.M. (2021). TGF-β signaling: From tissue fibrosis to tumor microenvironment. Int. J. Mol. Sci..

[36] Li Y., Wei Y., Tang W., Luo J., Wang M., Lin H., Guo H., Ma Y., Zhang J., Li Q. (2019). Association between the degree of fibrosis in fibrotic focus and the unfavorable clinicopathological prognostic features of breast cancer. PeerJ.

[37] Piersma B., Hayward M.K., Weaver V.M. (2020). Fibrosis and cancer: A strained relationship. Biochim. Biophys. Acta Rev. Cancer.

[38] Olivarez S.A., Maheshwari B., McCarthy M., Zacharias N., van den Veyver I., Casturi L., Sangi-Haghpeykar H., Aagaard-Tillery K. (2010). Prospective trial on obstructive sleep apnea in pregnancy and fetal heart rate monitoring. Am. J. Obstet. Gynecol..

[39] May A.M., Mehra R. (2014). Obstructive sleep apnea: Role of intermittent hypoxia and inflammation. Semin. Respir. Crit. Care Med..

[40] Chen Y.C., Chen T.W., Su M.C., Chen C.J., Chen K.D., Liou C.W., Tang P., Wang T.Y., Chang J.C., Wang C.C. (2016). Whole genome DNA methylation analysis of obstructive sleep apnea: IL1R2, NPR2, AR, SP140 methylation and clinical phenotype. Sleep.

[41] Nanduri J., Semenza G.L., Prabhakar N.R. (2017). Epigenetic changes by DNA methylation in chronic and intermittent hypoxia. Am. J. Physiol. Lung Cell Mol. Physiol..

[42] Zhu Z., Cao F., Li X. (2019). Epigenetic programming and fetal metabolic programming. Front. Endocrinol..

[43] Elshenawy S., Simmons R. (2016). Maternal obesity and prenatal programming. Mol. Cell Endocrinol..

[44] Hilakivi-Clarke L., de Assis S. (2006). Fetal origins of breast cancer. Trends Endocrinol. Metab..

[45] Pathiraja T.N., Nayak S.R., Xi Y., Jiang S., Garee J.P., Edwards D.P., Lee A.V., Chen J., Shea M.J., Santen R.J. (2014). Epigenetic reprogramming of HOXC10 in endocrine-resistant breast cancer. Sci. Transl. Med..

[46] Widschwendter M., Siegmund K.D., Müller H.M., Fiegl H., Marth C., Müller-Holzner E., Jones P.A., Laird P.W. (2004). Association of breast cancer DNA methylation profiles with hormone receptor status and response to tamoxifen. Cancer Res..

[47] Mabry S., Wilson E.N., Bradshaw J.L., Gardner J.J., Fadeyibi O., Vera E., Osikoya O., Cushen S.C., Karamichos D., Goulopoulou S. (2023). Sex and age differences in social and cognitive function in offspring exposed to late gestational hypoxia. Biol. Sex. Differ..

[48] Bin Y.S., Cistulli P.A., Roberts C.L., Ford J.B. (2017). Childhood health and educational outcomes associated with maternal sleep apnea: A population record-linkage study. Sleep.

[49] Tauman R., Zuk L., Uliel-Sibony S., Ascher-Landsberg J., Katsav S., Farber M., Sivan Y., Bassan H. (2015). The effect of maternal sleep-disordered breathing on the infant’s neurodevelopment. Am. J. Obstet. Gynecol..

[50] Casale M., Pappacena M., Rinaldi V., Bressi F., Baptista P., Salvinelli F. (2009). Obstructive sleep apnea syndrome: From phenotype to genetic basis. Curr. Genom..

