# Supplementary material for: Gestational Intermittent Hypoxia Enhances Mammary Stem Cells and Alters Tumor Phenotype in Adult Female Offspring
Source: Cells. 2024 Jan 29;13(3):249. doi: 10.3390/cells13030249 (PMC10854849; doi:10.3390/cells13030249)
Supplement: Supplementary file 1 [file cells-13-00249-s001.zip › Supplementary material/Supplementary Figure Legends.pdf]

Supplementary Figure Legends

Figure S1. Mammary gland development, timing of puberty and estrus cycle in offspring rats. Ductal outgrowth in mammary glands from GNx and GIH offspring rats at 8 weeks (A, n=6 rats/group) and 20 weeks (B, n=6 GNx, n=5 GIH rats). Alveolar budding in mammary glands of offspring rats at 8 weeks (C, n=6/group) and 20 weeks (D, n=6 GNx, n=5 GIH rats). (E) Day of vaginal opening in offspring rats (n=9/group). Length of time spent in estrus (F) or diestrus (G) during the estrus cycle of rats from the day of vaginal opening measured over 2 weeks (n=9/group).

Figure S2. Characterization of adipose-derived stromal cells from mammary glands of GNx and GIH offspring rats. Expression of epithelial cell marker CD24 (A), endothelial cell marker CD31 (B), and immune cell marker CD45 (C) in cultured adipose-derived stromal cells from 8-week-old rats quantified using qRT-PCR (n=4/group). Primary mammary epithelial cells (MEC) and spleen tissue were used as controls. (D) Expression of adipose-derived stromal cell markers CD49f, CD73, CD90, and CD105 in cultured cells (n=4/group).

Figure S3. Latency of tumors from GNx and GIH aging offspring rats. (A) Kaplan-Meier analysis of mammary tumor-free survival of offspring rats. (B) Percent of offspring rats that developed mammary tumors. (C) Percent of offspring rats that had both mammary and pituitary tumors. (D) Latency in months of mammary tumors that developed in rats that also had pituitary tumors. (E) Representative images and quantification of estrogen receptor alpha (ERα) expressing cells within tumors (n=5/group).
