# Supplementary material for: Gestational Intermittent Hypoxia Enhances Mammary Stem Cells and Alters Tumor Phenotype in Adult Female Offspring
Source: Cells. 2024 Jan 29;13(3):249. doi: 10.3390/cells13030249 (PMC10854849; doi:10.3390/cells13030249)
Supplement: Supplementary file 1 [file cells-13-00249-s001.zip › Supplementary material/Table S1.pdf]

Table S1. Primers used for quantitative RT-PCR analysis.

Gene	Forward Primer	Reverse Primer
Pparγ	CACAAGAGCTGACCCAATGGT	GGCTCTTCATGTGGCCTGTT
Fabp4	AGCTGGTGGTGGAATGTGTC	ATTTCAGTCCAGGGCCTCGT
Alpl	AATCGGAACAACCTGACTGACCCT	AATCCTGCCTCCTTCCACTAGCAA
Runx2	GCGTCAAACAGCCTCTTCAGT	GCTCGCAGCACACTACCCTT
Cd24	GATTGGAACCCACCCCCAAA	CCTTGCTAACAGACCACGCT
Cd31	CAGCCATTACGACTCCCAGA	GAGCCTTCCGTTCTCTTGGT
Cd45	GGAAGGCACTCGGACTTTCA	TCCGTCGAAGTTTCAGGAGC
18s	CGGGTGCTCTTAGCTGAGTGTCCC	CTCGGGCCTGCTTTGAACAC
Cd49f	GATGCCACCTACCACAAGGC	TTTCATTCCACTTCGGGGGC
CD105	CTCCAGGCATCCAACACCAT	TGTGGGCACCATGTAGACAC
CD73	TGCATCGATATGGCCAGTCC	AATCCATCCCCACCGTTGAC
CD90	ACCAAGAGGAGGCTGCAAG	CTTCTTCTCTCGGGTCAGGC
