# Supplementary figures and images for: Gestational Intermittent Hypoxia Enhances Mammary Stem Cells and Alters Tumor Phenotype in Adult Female Offspring
Source: Cells. 2024 Jan 29;13(3):249. doi: 10.3390/cells13030249 (PMC10854849; doi:10.3390/cells13030249)

Figure S1

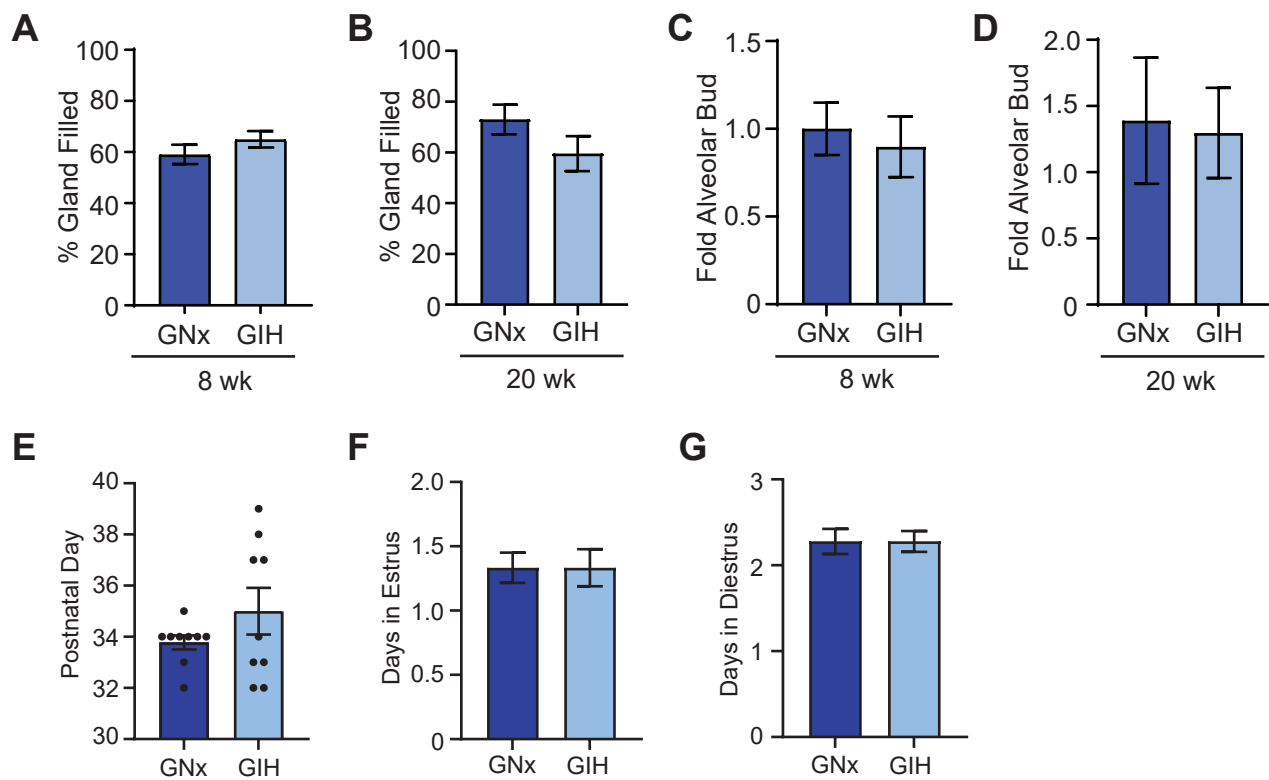

Supplement: Supplementary file 1 [file cells-13-00249-s001.zip › Supplementary material/Fig S1.pdf]

Figure S2

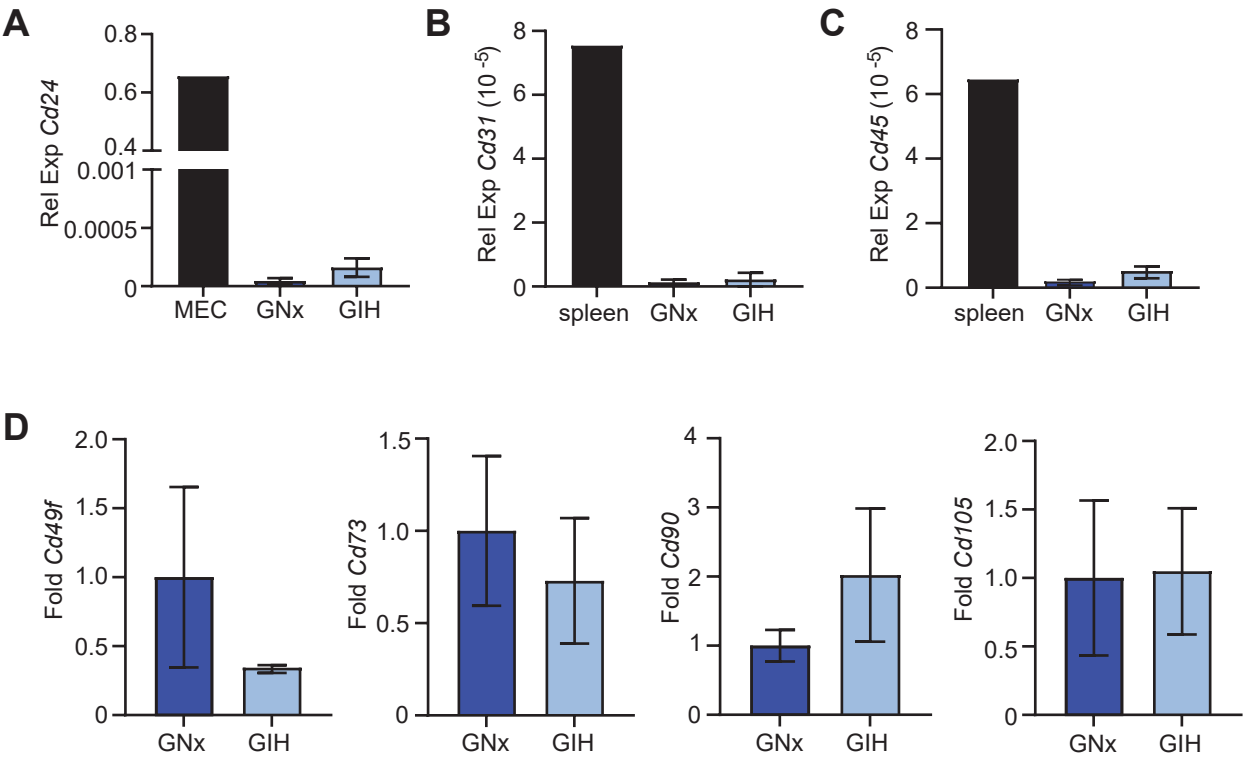

Supplement: Supplementary file 1 [file cells-13-00249-s001.zip › Supplementary material/Fig S2.pdf]

Figure S3

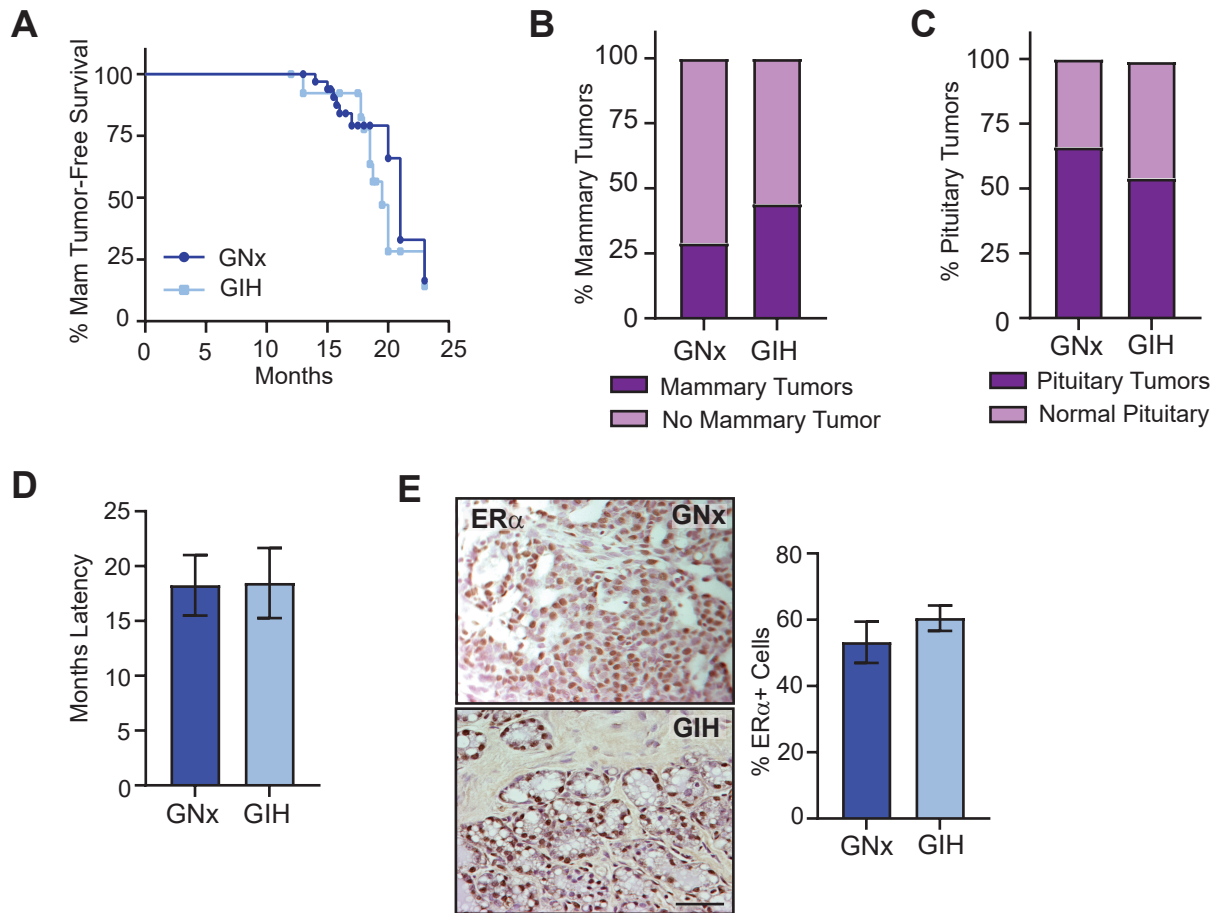

Supplement: Supplementary file 1 [file cells-13-00249-s001.zip › Supplementary material/Fig S3.pdf]
